# Functional and Quantitative MRI Mapping of Somatomotor Representations of Human Supralaryngeal Vocal Tract

**DOI:** 10.1093/cercor/bhw393

**Published:** 2017-01-09

**Authors:** Daniel Carey, Saloni Krishnan, Martina F. Callaghan, Martin I. Sereno, Frederic Dick

**Affiliations:** 1 Department of Psychology, Royal Holloway, University of London, London, TW20 0EX, UK; 2 The Irish Longitudinal Study on Ageing, Department of Medical Gerontology, Trinity College Dublin, Dublin 2, Ireland; 3 Department of Psychological Sciences, Birkbeck College, University of London, Malet St, London, WC1E 7HX, UK; 4 Department of Experimental Psychology, Tinbergen Building, 9 South Parks Road, Oxford, OX1 3UD, UK; 5 Wellcome Trust Centre for Neuroimaging, Institute of Neurology, University College London, 12 Queen Square, London, WC1N 3BG, UK; 6 Birkbeck/UCL Centre for Neuroimaging, 26 Bedford Way, London, WC1H 0AP, UK; 7 Department of Experimental Psychology, UCL Division of Psychology and Language Sciences, 26 Bedford Way, London, WC1H 0AP, UK; 8 Department of Psychology, College of Sciences, San Diego State University, 5500 Campanile Drive, San Diego, CA 92182-4611, USA

**Keywords:** mapping, MRI, somatomotor, speech, vocal tract

## Abstract

Speech articulation requires precise control of and coordination between the effectors of the vocal tract (e.g., lips, tongue, soft palate, and larynx). However, it is unclear how the cortex represents movements of and contact between these effectors during speech, or how these cortical responses relate to inter-regional anatomical borders. Here, we used phase-encoded fMRI to map somatomotor representations of speech articulations. Phonetically trained participants produced speech phones, progressing from front (bilabial) to back (glottal) place of articulation. Maps of cortical myelin proxies (R_1 _= 1/T_1_) further allowed us to situate functional maps with respect to anatomical borders of motor and somatosensory regions. Across participants, we found a consistent topological map of place of articulation, spanning the central sulcus and primary motor and somatosensory areas, that moved from lateral to inferior as place of articulation progressed from front to back. Phones produced at velar and glottal places of articulation activated the inferior aspect of the central sulcus, but with considerable across-subject variability. R_1_ maps for a subset of participants revealed that articulator maps extended posteriorly into secondary somatosensory regions. These results show consistent topological organization of cortical representations of the vocal apparatus in the context of speech behavior.

## Introduction

The supralaryngeal vocal tract (SVT) comprises a complex set of sensory surfaces and motor effectors that, in primates, are represented within and across multiple cortical areas. In macaques, single-cell recording evidence indicates overlapping representations of jaw and tongue movements moving inferiorly in primary motor cortex (M-I); upper lip, lower lip, teeth, and tongue also show a superior to inferior mapping in primary somatosensory cortex (S-I) (Huang et al. 1988, 1989a, 1989b; Murray and Sessle 1992a, 1992b; see also Arce-McShane et al. 2014). Such functional representations overlay cyto- and myeloarchitectonically differentiated cortical areas; for instance, within the elongated and rostral-bending area 3B in owl and squirrel monkeys, there are multiple myelin-dense patches that correspond with electrophysiological mappings of the lips, teeth, and tongue (Jain et al. 2001). However, differences in coverage among species, differences in cortical folding patterns, and marked interspecies differences in vocal capabilities make generalizations across primates challenging, particularly when extending such findings to humans (Sherwood et al. 2004; Petrides et al. 2005; Kumar et al. 2016; see also Fitch 2000; Ackermann et al. 2014).

The arrangement and nature of representations within somatomotor regions remain topics of debate. While the traditional accounts of Penfield and colleagues indicated separable effector musculotopy, more recent models have suggested additional topological maps of movement classes (Aflalo and Graziano 2006a; Graziano and Aflalo 2007). For instance, evidence from nonhuman primates has shown that complex, multi-effector forelimb postures (Graziano et al. 2002a, 2002b; Aflalo and Graziano 2006b; Overduin et al. 2012) and oral effector movements (Graziano et al. 2002a) occur following electrical stimulation of M-I neurons. Moreover, such movements typically occur toward specific locations in space (Graziano et al. 2005; Overduin et al. 2012), are evoked at timescales relevant to performing complex actions (Graziano et al. 2002a), and largely fall within the set of ethological activities relevant to the animal (e.g., feeding and self-defense; Graziano and Cooke 2005). These studies have emphasized the role of somatomotor representations in controlling an ensemble of effectors in the service of a specific behavior. While such questions have been addressed extensively in primates and with respect to manual movement, considerably less is known about oral movements, particularly in humans. The complexity of human oral behaviors such as speech articulation motivates exploration of the nature of M-I and S-I representations of the SVT with respect to that behavior. Indeed, speech necessitates not only the contact between the SVT articulators, but also complex synergies of effector movements, careful planning before and following each articulation, and control of airflow mechanisms.

To date, a number of human fMRI studies have compared activation for movements of the lips, tongue, and jaw. Hesselmann et al. (2004) compared lip pursing and “horizontal tongue excursions” in blocks; within the central sulcus they found that preferential activation for lip pursing lay superior to that for tongue movements, with the latter activation greatest near the base of the central sulcus. This held true bilaterally in group maps as well as in the position of peak activations for each subject. A similar somatotopy related to backward-forward tongue movement and lip pursing was seen in all individual as well as group “winner-take-all” motor movement maps of Meier et al. (2008). Using tactile stimulation alone of the right tongue, upper incisor, and lower lip, Miyamoto et al. (2006) showed an inferior to superior progression of preferential activation for these surfaces within approximately the same region of central sulcus, with a somewhat more mosaic arrangement posteriorly. Hesselmann et al. (2004), Miyamoto et al. (2006), and Meier et al. (2008) emphasize the high degree of overlapping activation for movements within these regions. Grabski et al. (2012b) compared activation for blocks of lip protrusion, tongue retraction, and jaw lowering, and showed greater activation in inferior parts of the central sulcus for tongue retraction versus either lip protrusion or jaw lowering, with no difference in activation between jaw and lip movement. By contrast, Grabski et al. (2012a) used a very similar paradigm within a single “sparse-sampling” run, and found no clear somatotopic arrangement at a group level, although with some somatotopic spread at an individual level across peak coordinates. In a study focused on characterizing laryngeal representations, Brown et al. (2008) visually compared activation peaks (vs. rest) for glottal stops, lip puckering, singing 5 scale notes with a schwa, and moving the tongue up and down (alternately contacting the hard palate and lower postdental ridge). Brown et al. (2008) described shared activation peaks for glottal stops and singing schwa that lay superior to the peaks for the lips and the tongue. Brown et al. (2009) statistically compared activation related to blocks of reciting Beowolf (with restricted jaw movement) to the same lip, tongue, and singing movements; critically, however, these comparisons revealed very limited somatotopic mapping of the articulators.

In sum, the majority of the studies above have employed traditional block-design contrasts of vocal tract stimulation conditions versus rest, or versus each other (but see Meier et al. 2008), which in some cases has revealed differences in somatomotor activation peaks for distinct effector movements (e.g., larynx, lips, and tongue; Brown et al. 2008). However, these analyses do not speak directly to the detailed topography of cortical representations that may arise due to the distinct positions and dynamics of the articulators during speech behavior (Sörös et al. 2006; Brown et al. 2009; see also Sato et al. 2014). Moreover, such cortical representations may not be adequately captured by activation loci that reflect mean BOLD signal change alone (Grabski et al. 2012a).

Recent advances in electrocorticography (ECoG) methods with presurgical patients have revealed additional details of the spatial and temporal dynamics of speech articulator representations (Bouchard et al. 2013; Bouchard and Chang 2014). Bouchard et al. (2013) showed that cortical activity for articulation of speech phones at labial (e.g., /ba/), alveolar (e.g., /da/), and palatal (e.g., /ga/) places followed a lateral to ventral gradient across electrodes that covered the lower half of somatomotor cortex. Further, a nearest-neighbor spatial clustering analysis showed that across speech phones involving a specific articulator (e.g., lips, tongue, or larynx), activity followed a broadly somatotopic gradient that emerged laterally to ventrally as larynx, lips, jaw, tongue, and larynx (Bouchard et al. 2013). Moreover, responses across electrode sites showed high temporal specificity, such that activity for production of consonants (e.g., plosives) consistently preceded activity for tongue height or backness associated with the vowel that followed (Bouchard et al. 2013; see also Bouchard and Chang 2014). While these ECoG findings break further ground in charting human vocal tract somatomotor representations, the placement of ECoG electrode arrays is largely restricted to recording sites on gyral crowns. This can lead to difficulty in localizing activity arising from locations only a few millimeters down into sulci, since current dipoles there will generate surface maxima and minima with substantial tangential displacement along the cortical surface (Dale and Sereno 1993; their Fig. 4A). As a result, the likelihood that articulatory cortical activity measured at the pial surface can be ascribed to its true source(s) is reduced. This is a key limitation of EcoG methods that do not explicitly model cortical radial source geometry, and presents an obstacle to accurately localizing articulator representations spread across gyral as well as sulcal regions, likely involved in articulatory behavior (see Guenther and Vladusich, 2012; further to Meier et al. 2008). Marked interindividual variation in cortical folding patterns and subject-wise variation in electrode array placement exacerbate this problem. The invasive nature of ECoG methods is not appropriate for mapping vocal tract somatomotor representations in the typical population. Finally, ECoG methods typically do not afford whole-brain coverage. fMRI mapping and cortical surface reconstruction offer a solution to all these difficulties, by allowing normal subjects’ maps to be averaged in a common spherical surface-based co-ordinate system (Fischl et al. 1999).

The detailed arrangement of articulator representations within and beyond human M-I/S-I thus remains unclear (Huang et al. 2012, on primary and extra-primary body surface representations, and Krippl et al. 2015, on facial movement). For example, we do not know their detailed internal spatial order, the number of possible rerepresentations of these surfaces, their bilaterality, or the degree to which brain regions involved in articulation are coextensive with these topological maps. Moreover, it is not clear how consistent these representations are within and between individuals—to date, fMRI studies have largely focused on cohort-level cortical responses, with limited consideration of inter or intrasubject variability (or indeed, stability). Finally, it is not known to what extent articulator representations are consistent across different manners of articulation, or how these functional representations relate to putative myelination differences that are associated with motor and somatosensory areal borders. A clearer understanding of vocal tract representations in the cortex is of general importance in charting the neural bases of speech production. Moreover, detailed vocal tract representational maps may prove to be of great utility in determining somatomotor representations associated with atypical speech in developmental disorders (particularly articulation difficulties, which are typically diagnosed via the Diadochokinetic rate; Henry 1990), or following brain injury (e.g., stroke).

Here, we used a phase-encoded fMRI design to map places of articulation across cortex using a phonetically trained cohort of experienced subjects. Testing a cohort that was experienced in phonetics (and in being scanned) helped to ensure that articulations were performed in a systematic fashion both across and within individuals. By measuring cortical responses that showed consistent amplitude and phase of the BOLD signal during articulation at specific places, we mapped SVT dynamics across both hemispheres, and compared the resulting maps to the broader cortical territory associated with activation for repeated articulation regardless of effector. Phase-encoded methods are often used for topographic functional mapping studies because they tend to produce robust results in a limited amount of scanner time (Engel 2012). Such phase-encoded or “cyclic” experimental designs may also reveal maps that would be very difficult to uncover using block or event-related designs; the increased efficiency of phase-encoded designs is likely due in part to the suppression or saturation of nonstimulus-specific BOLD responses due to continuous stimulation (Moon et al. 2007). The difference in efficiency between the 2 experimental designs may help to explain why several previous studies using block-design contrasts have shown very little evidence of distinctions in somatomotor representations between specific articulators (Grabski et al. 2012a; see also Sörös et al. 2006). However, to relate our findings to previous work, we also performed block design experiments in a subset of our participants to illustrate the full extent of regions involved in articulatory behavior (contrasting articulation of groups of phones vs. rest).

In a subset of participants, we further compared phase-encoded fMRI maps to high-resolution quantitative MR scans that provide a proxy measure for cortical myelin (R_1_ = 1/T_1_). This made it possible to relate functional map boundaries to changes in putative myelination associated with somatomotor areal boundaries in a more precise manner, since transitions between primary and nonprimary cortex often do not reflect gross gyral or sulcal landmarks (Sereno et al. 2013; Glasser et al. 2016). While probabilistic atlases can allow estimation of inter-regional boundaries (Eickhoff et al. 2005; see also Glasser and Van Essen, 2011), we were able to directly situate articulator maps with respect to subject-specific in vivo anatomical proxies for primary regions (M-I/S-I).

Finally, as a test of how specific the phase-encoded maps were to the manner of articulation (see also Correia et al. 2015; Cheung et al. 2016), we generated maps in a subset of our participants for production of fricatives that varied in place of articulation in a similar manner to the voiceless stops used in the main experiment.

## Materials and Methods

### Participants

Participants were 10 healthy adults (mean age = 34.2 years; Standard Deviation [SD] = 10.77; age range = 22–57; 4 males, 6 females). Participants were recruited from the School of Psychological Sciences, Birkbeck College and the School of Speech, Hearing and Phonetic Sciences, University College London. Handedness was recorded by self-report, with 9 participants right-handed and one left-handed (The left-handed participant was included since we had no reason to assume that elementary vocal tract representations within M-I and S-I would differ as a function of handedness—indeed, the results for this subject supported this conclusion). Overall, 7 participants had phonetic training (mean = 6.43; SD = 5.53 years of training). The remaining participants had extensive experience with language research and had practiced producing the stimuli in advance of the experiment. Seven participants were native English speakers and spoke with a variety of dialects (British, North American, Irish); the remaining 3 spoke English to native level proficiency, with British or American dialects. All but one participant had learned multiple languages other than English. Factors such as language history can affect sensorimotor control in the context of articulation, and so it is important to control for and minimize such effects. We sought to ensure consistency in articulatory performance by testing subjects with training in phonetics and/or extensive practice in producing controlled oral motor movements. We expand on this point. In the present study, the units of articulation were isolated voiceless stops or fricatives, and were drawn from a number of different language families. Two of the phones in the main experiment (retroflex - /ʈə/, palatal /cə/) were non-native to several of our subjects. The training in phonetics our subjects had (in addition to all subjects practising the phones before scanning) helped to ensure that the impact of language background was minimized as much as possible during articulation. For example, S6 (Fig. 2*f*) is a native English speaker and an experienced scientist without training in phonetics who was well-practiced at the tasks, while S10 (Fig. 2*j*) is also a native English speaker and a lecturer in phonetics; comparison of the maps for these subjects suggests very close correspondence. Similarly, S5 (Fig. 2*e*) is a native English speaker with training in phonetics, while S8 (Fig. 2*h*) is a non-native English speaker and a lecturer in phonetics; again, close correspondence of maps is observed between these 2 subjects. Our cohort also varied in age range: 3 subjects in the current cohort were over the age of 40 years at the time of the study, and 1 was at the upper age in the range (57 years old). Two of these 3 (including the eldest subject) have undergone audiometric screening since the study and had pure tone thresholds in the normal range. All of our subjects have experience of working within speech and language research; none reported any hearing difficulties (e.g., tinnitus and hearing loss) nor related speech or language issues (e.g., difficulty perceiving speech in noise). The study received ethical approval from the local ethics committee, and participants provided voluntary informed consent prior to commencing.

**Figure 1.**
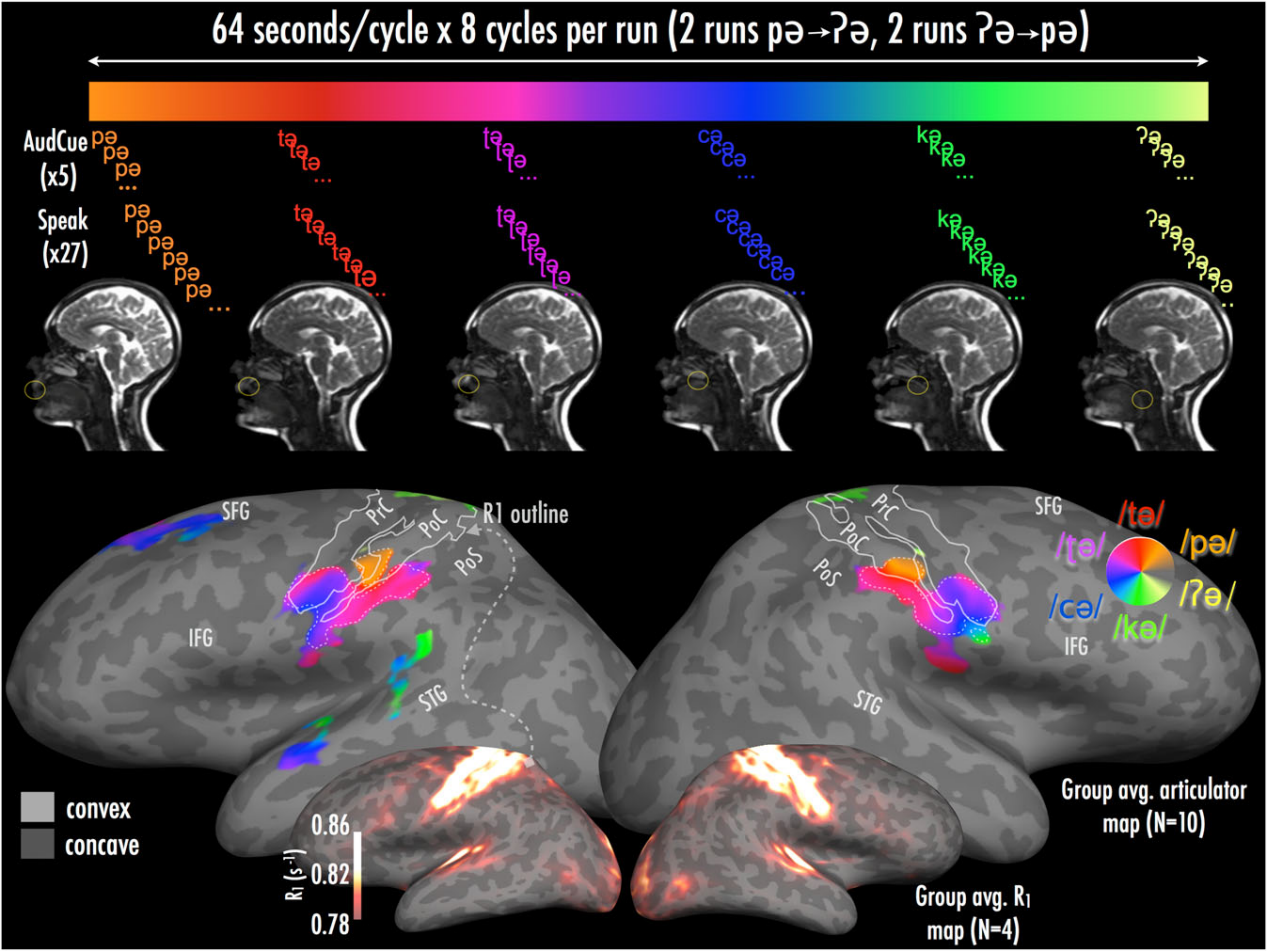
(Top panel) Schematic of a single 64-s cycle of main articulation experiment. Midsagittal view shows True FISP image acquired while participant S6 performed each articulation; circle indicates approximate place of articulation. (Bottom panel) Group average phase-encoded articulator maps displayed with cluster-corrected (281 mm^2^) significance of *P* < 10^–5^ (initial uncorrected threshold *P* < 0.05). Map boundaries at a more conservative threshold (initial uncorrected *P* < 0.01, cluster size 86 mm^2^, corrected hemisphere-wise significance *P* < 0.001) are shown as the dashed trace on top of the *P* < 10^−5^ cluster-corrected map. Data are displayed on a single participant's inflated cortical surface, where color hue shows significant periodic responses across cortical areas at a given stimulus frequency (i.e., the phase of the response); color saturation indicates the magnitude of the periodic response (Sereno et al., 1995; Saygin and Sereno 2008; Huang et al. 2012). Color-coding of periodic response at the stimulus frequency for each plosive is shown in a counter-clockwise direction on the graded phase wheel, from anterior to posterior places of articulation. Inlaid inflated surfaces with heatscale overlay show R1 values for the cortical-surface-based average of participants S1, S2, S6, and S7, with values sampled at 0.5 cortical depth fraction. The R1 contour (solid trace) overlaid on the articulation maps was drawn from this average map at a threshold of R_1_ ≥ 0.82 s^–1^, and approximates the borders of the heavily myelinated area 4 anteriorly, and 3b posteriorly. Abbreviations: PrC—precentral gyrus; PoC—postcentral gyrus; PoS—postcentral sulcus; STG—superior temporal gyrus; SFG—superior frontal gyrus; IFG—inferior frontal gyrus.

Stimuli: In the main fMRI experiment, participants were auditorily prompted to produce trains of the following voiceless stops (plus neutral schwa vowel) that varied systematically in their place of articulation: bilabial (/pə/); alveolar (/tə/); retroflex (/ʈə/); palatal (/cə/); velar (/kə/); glottal (/ʔə/). (See Fig. 1 and AudioFile1). Bilabial stops (initial consonant in English “pea”) involve rapid opening of the lips in tandem with a small downward movement of the lower jaw. Alveolar stops (English “ta”) require contact and quick release of the tongue tip or blade with the alveolar ridge. In retroflex stops (Indian English “time”), the tongue tip is curled back and contacts the postalveolar area before release. Palatal stops (Italian “chi”) instead require the middle or back part of the tongue to contact the hard palate, whereas in velar stops (English “kiss”) the back of the tongue contacts the soft palate. Glottal stops (English “uh”) involve glottal closure without tongue movement.

Prompts were excised from a recording of a phonetically trained female native English speaker producing each plosive. Stimuli were scaled to a nominal intensity of 65 dB RMS in Praat (Praat, 5.3.01), and inspected to ensure peak clipping of sounds had not occurred. A token of each stop + vowel (padded by 40 ms of silence, total duration 330 ms) was concatenated 5 times to create a prompt train, with a syllable repetition rate of 3 Hz. Auditory prompts were then concatenated in order of place of articulation, either front-to-back (/pə/ /tə/ /ʈə/ /cə/ /kə/ /ʔə/) or the reverse, with each prompt separated by 9 s of silence, during which the participant repeated the prompted syllable at the same rate (see schematic in Fig. 1). The full cycle of prompts repeated every 64 s, with 8 front-to-back or back-to-front cycles per run (run duration 8′32″). Stimuli were presented binaurally using Sensimetrics S14 earbuds.

Procedure: Prior to scanning, the experimenters familiarized all participants with the syllabic prompts (typically, for 20–30 min), until participants could produce them at the required rate without difficulty. Care was taken to ensure all participants could perceive the speech sounds clearly and could produce all stops with the correct place of articulation. Critically, participants were instructed to reduce movement of the jaw and lips during production in order to minimize head movement artifacts. Participants also practiced producing each sound with as soft an articulation as possible to reduce artifact arising from B0 field distortions due to changes in tongue position and air volume within the mouth, throat, and chest. Participants were instructed to produce each speech sound at a constant rate, starting immediately at the onset of the prompt and at the prompted tempo (3 syllables/s). Participants continued producing the prompted sound after offset of the prompt, and began production at the next place of articulation at the next prompt onset (10.66 s after onset of the previous prompt). The stimulus prompt sound file began playing at the beginning of the fifth TR of each functional scanning run (the first 4 images were discarded to allow T_1_ magnetization to come to equilibrium). Participants underwent 2 alternated sessions of each place of articulation order (front to back and back to front). Participants were monitored by in-bore video camera and encouraged to breathe at irregular intervals during functional scanning, to reduce potential data artifact arising from systematic breathing and/or head movement.

Data acquisition: Functional data were acquired on a 1.5 T Siemens Avanto scanner with 32-channel head coil using a T_2_*-weighted echo planar gradient echo pulse sequence (256 TRs, TR = 2000 ms, TE = 39 ms, flip angle = 90°, bandwidth = 1474 Hz/pixel, matrix = 64 × 64, 24 axial slices, 3.2 × 3.2 × 3.2 mm^3^ voxels). The first 4 volumes in each run were excluded to allow for T_1_ longitudinal magnetization to reach steady state. A T_1_-weighted magnetization-prepared rapid gradient echo (MPRAGE) scan was acquired for each subject (TI = 1000 ms, TR = 8.4 ms, TE = 3.57 ms, flip angle = 7°, matrix = 224 × 256, 176 axial slices, 1 × 1 × 1 mm^3^ voxels). A multiparameter mapping (MPM) protocol (Weiskopf et al. 2013; Lutti et al. 2014) was also acquired on 4 of the participants. Proton density-weighted (PDw), T1-weighted (T1w), and magnetization transfer (MTw) images were acquired using an in-house 3D FLASH pulse sequence (voxel size: 0.8 × 0.8 × 0.8mm^3^, FOV = 256 × 224 × 180mm^3^, matrix = 320 × 256 × 224, TR = ×16.0ms, bandwidth 480 Hz/px, excitation flip angle: 4° (PDw/MTw) or 24° (T1w), slab rotation 30°). To accelerate this high-resolution acquisition, a partial Fourier acquisition (6/8 coverage) was used in the inner phase-encoded direction (RL) and parallel imaging was used along the outer phase encoding direction (AP), reconstructed using the GRAPPA algorithm (acceleration factor 2, 32 integrated auto-calibration lines) as implemented on the scanner platform. Four gradient echoes were acquired for each contrast (TE = 2.50, 4.84, 7.18, 9.52 ms) after each RF excitation pulse and averaged to improve SNR (Helms and Dechent 2009). Quantitative R_1_ (=1/T_1_) maps were estimated from the PDw and T1w images according to the model developed by Helms et al. (2008) which was extended by including a correction for RF transmit field inhomogeneities (Lutti et al. 2010) and imperfect spoiling (Preibisch and Deichmann 2009). The transmit field map was calculated using a 3D EPI spin-echo (SE)/stimulated echo (STE) method (Lutti et al. 2010; Lutti et al. 2012; FOV = 256 × 192 × 192 mm^3^, matrix = 64 × 64 × 48, TE/TM = 50.02/44.16 ms, TR = 500 ms, nominal α varying from 115° to 65° in steps of 5°, acquisition time 4 min 24 s) and was corrected for off-resonance effects using a standard B0 field map (double gradient echo FLASH, 3 × 3 × 2 mm^3^ isotropic resolution, whole-brain coverage).

Data processing, structural scans: The MPRAGE anatomical scans were used to reconstruct the cortical surface of 6 participants (Dale and Sereno 1993; Dale et al. 1999; Fischl et al. 1999), except for the 4 participants who underwent the MPM protocol, in which case these scans were used for surface reconstruction (Lutti et al. 2014). A 6-subject cortical-surface-based average group R_1_ map, corrected for local effects of cortical thickness and curvature (Dick et al. 2012; Sereno et al. 2013) was spherically morphed to the display subject's brain for comparison with articulation maps. (The 2 subjects from the current study were included in the Sereno et al. 2013 data.) The gradient of the group R_1_ map was also overlaid with articulation maps to estimate the borders of secondary somatosensory areas (Glasser and Van Essen 2011).

Data processing, functional scans. All functional data were analyzed using a customized version of FreeSurfer (csurf, M. Sereno, http://www.cogsci.ucsd.edu/~sereno/.tmp/dist/csurf). Functional data were registered using a linear affine least-squares minimization algorithm in AFNI (3dvolreg; Cox 1996); functional scans were then registered using bbregister (Greve and Fischl 2009) and manual blink comparison to the high-resolution volume used to create the cortical surface (i.e., rapidly flicking between functional volumes and the anatomical image in csurf tkregister to check registration success).

Each functional session was analyzed using Fourier analysis methods (Sereno et al. 2013), where functional activation is measured as the amplitude of the periodic BOLD signal at the frequency of the stimulus cycle (Hagler et al. 2007). Periodic signal components with very low frequencies (due to motion) and the second and third harmonic of the stimulus (due to surround inhibition), as well as the higher frequency of the auditory prompt, were excluded as neither signal nor noise (this is mathematically equivalent to first linearly regressing out these frequencies as nuisance variables before calculating significance).

For each subject, the full Fourier transforms of each functional run time course were calculated, with the calculated phase subsequently reversed at the stimulus (but not noise) frequencies for the back-to-front runs. A complex *F*-ratio was then calculated by comparing the Fourier amplitude at stimulus frequencies to the average Fourier amplitude at nonstimulus frequencies (Hagler et al. 2007; Saygin and Sereno 2008). Averaged 3D Fourier amplitudes and first level statistics were painted onto each participant's inflated cortical surface in csurf.

A cross-subject activation average was created using spherical-registration-based cross-subject resampling (Fischl et al. 1999a; Hagler et al. 2007). Each subject's statistical maps were resampled onto the icosahedral spherical surface using best-fit sulcal alignment with one step of surface-based smoothing. Group-level statistics were then calculated via a cross-subject *F*-ratio (based on the complex Fourier coefficients at the stimulus frequency from each subject) with (2, 2*n*–2) degrees of freedom. Averaged data were resampled onto a single subject's surface for visualization displayed with 10 steps of surface-based smoothing (approximating a Gaussian smoothing kernel of 3 mm FWHM). Surface-based cluster exclusion was used to correct for multiple comparisons (surfclust and randsurfclust from Hagler et al. 2006, 2007); group-level *F* statistics were thresholded at *P* < 0.05 and surface clusters less than 281 mm^2^ excluded (achieving a cluster-corrected significance of *P* < 10^−5^ per hemisphere). Surface cluster threshold extent was determined based on the minimum estimated cortical area from iterative random sampling of cluster sizes (*N* = 100 000 iterations per hemisphere by randsurfclust; Hagler et al. 2006, 2007) that were required to achieve a corrected alpha of *P* < 10^−5^ for each hemisphere, based on an initial uncorrected alpha of 0.05. Additionally, we ensured robustness of results at a more conservative initial threshold of *P* < 0.01 (cluster sizes 86 mm^2^, calculated with 10 000 iterations per hemisphere, achieving corrected hemisphere-wise alpha of 0.001). We additionally present results with less conservative cluster correction in supplementary figure 1 (initial uncorrected alpha 0.05, cluster size 134 mm^2^, corrected hemisphere-wise alpha 0.05).

### Additional Experiments

To further explore the phase-encoded results from the main experiment, we conducted a series of additional experiments with the most experienced scanner subjects from the main experiment. Those subjects had shown clear maps of most of the voiceless stops in the main experiment, were adept at remaining still throughout extended scanning sessions, and showed good tolerance of the challenging experimental protocol. This follows previous cortical mapping studies that have conducted control conditions using subsets of their full sample (Saygin and Sereno 2008; Pitzalis et al. 2010; Huang et al. 2012).

1) Articulator mapping using unvoiced fricatives: S6 and S7 completed an additional four-run experiment where 9 different unvoiced fricatives were articulated at a slightly faster rate of ~4.5 Hz. As in the main experiment, a short train of syllables was played to cue the participant when the place of articulation changed (every 7.11 s). In order of presentation (with IPA symbol, place of articulation, and example word containing sound), these were: /ɸ/ bilabial (final fricative in German “Topf”); /f/ labiodental (English “fix”); /θ/ dental (English “thin”); /s/ alveolar (English “sit”); /ʃ/ palato-alveolar (English “ship”); /ʂ/ retroflex (Polish “szum”); /ç/ palatal (German “nicht”); /x/ velar (German “Buch”); /h/ glottal (English “hat”). Data acquisition and processing were the same as the main experiment.

2) Experiments contrasting articulation versus resting baseline, or alveolar versus glottal voiceless stops: S1, S2, S6, and S7 took part in 2 additional block design experiments in a single scanning session. Each experiment consisted of 2 runs of 4 min (120 TRs each run), presented in the same order for all participants. At the beginning of each block, participants heard an auditory prompt (5 repetitions of a syllable like the main experiment) which cued them to articulate that sound until the onset of the next block, when they heard a different syllable cue, or an instruction to “rest.” In the first experiment, subjects articulated 11-to-15 s blocks of bilabial /pə/, alveolar /tə/, retroflex /ʈə/, palatal /cə/, or velar /kə/, with each articulation block followed by a 10-s rest block; time allocated to each place of articulation was balanced over the 2 runs. In the second experiment, participants were prompted to articulate alveolar /tə/ or glottal /ʔə/ in 11–12-s blocks, or to rest (10 s). In both experiments, block order was pseudorandomized such that each articulation type was repeated the same number of times over the experiment, and that the one block of a given articulation type did not follow itself.

Each participant's data were preprocessed and analyzed in FSL 5.0.8. Functional data were motion-corrected using MCFLIRT (with manual cine inspection of timeseries) slice-timing corrected, deskulled, temporal high-pass filtered (90 s cutoff), and registered to a single functional align volume from the same session. No spatial smoothing was used. The experimental design was temporally filtered and convolved with a gamma function to simulate the hemodynamic response function. For the first experiment, a single contrast was calculating comparing all articulation types versus rest; for the second, alveolar, and glottal stops were separately contrasted to rest, and to each other.

For each participant, the EPI align volume was registered using bbregister (Greve and Fischl 2009) to the high-resolution T1 volume used to reconstruct the participant's cortical surface. The parameter estimates and variance estimates for each contrast were resampled to the cortical surface, and then morphed based on sulcal and gyral alignment patterns to the unit icosahedron (Fischl et al. 1999) for cross-subject fixed effects analysis using mri_glmfit in Freesurfer.

## Results

Here, we used a phase-encoded fMRI paradigm to noninvasively map the vocal tract articulators, as subjects produced a range of speech phones that varied systematically in their place of articulation. We situated these phase-encoded maps with respect to quantitative MR markers (R_1_ = 1/T_1_) that provide putative “myelination maps” that can be used to estimate the extent of somatomotor cortical areas (Glasser and van Essen 2011). To orient maps with respect to canonical activation during articulation, we further compared phase-encoded maps for a subset of the subjects to block-design fMRI data collected as those subjects articulated the same speech phones in the “articulation versus rest” experiment. Finally, to determine how specific the phase-encoded maps were to the manner of articulation employed, we examined articulator maps in a subset of our participants for the articulation of fricatives that varied in place of articulation (vs. the voiceless stops used in the main experiment).

Group average (Fig. 1*a*): Somatomotor representation. The group average phase-encoded map shows medial (superior) to lateral (inferior) mapping across the unfolded somatomotor cortical areas as participants articulated consonants, with place of articulation progressing from anterior to posterior inside the mouth. The articulatory mapping was quite similar in both hemispheres, with slight differences noted below.

Articulation of bilabial stops (i.e., the most anterior place of articulation in the mouth) produced the most medial responses in the central sulcus (orange), which were concentrated in a compact ~1 cm patch that extended onto the postcentral gyrusjust lateral (inferior) to the characteristic posterior bend in the central sulcus caused by the motor cortex “hand knob.”

The alveolar response (red) adjoined the bilabial response region as a continuous thin inverted U-shaped stripe that extended from the anterior bank of the precentral gyrus, into the fundus of the central sulcus. The alveolar response then bent superiorly (inferior to the bilabial mapping), and then extended out onto the postcentral gyrus, where an additional leg of the inverted U extended for some distance medially into the postcentral sulcus (we note also that at a more conservative initial threshold (*P* < 0.01, Fig. 1, dashed trace), there was a small discontinuity at the left hemisphere between the bilabial and alveolar responses at postcentral gyrus). There was also some alveolar mapping in presumptive S-II just inferior and posterior to area 3b; this was more evident in the individual subject maps discussed below.

Activation to retroflex stops (magenta) covered a larger cortical region inferior to the alveolar-stop-related activation, with a similar shape. The top of the retroflex inverted U lay in the central sulcus, with the sides coming up onto the pre and postcentral gyri, with a similar medial extension into the postcentral sulcus, as well as an extension into presumptive secondary somatosensory areas inferior and posterior to the maximal alveolar response.

Just inferior to the retroflex region, the similarly shaped palatal stop response (blue) was more confined to the central sulcus, extending less onto the pre and postcentral gyri (a small discontinuity between the retroflex and palatal responses emerged at the right hemisphere at the more conservative initial threshold, *P* < 0.01—Fig. 1, dashed trace). Much more superiorly within the postcentral sulcus, there was a disconnected region of palatal response adjoining the medial retroflex representation.

Velar stops (green) occupied the smallest extent in the group average (but see individual maps, below), and were located just inferior to the palatal representation in the central sulcus, extending slightly onto the subcentral gyrus; there was a smaller response in the left homologue (although clearly present in a number of individual participants). There was also a small unexpected response in the central sulcus near the midline in a region typically associated with representations of the trunk or foot. Finally, we did not observe any significant group somatomotor representation uniquely associated with glottal stops (yellow), a point we return to below.

Responses outside traditionally defined somatomotor regions: There were isolated patches showing periodic responses during retroflex and palatal stops in multiple left and right hemisphere regions previously associated with auditory language comprehension: the superior temporal gyrus and sulcus (including one very posterior patch in a part of the presumptive angular gyrus default-mode region, just inferior to higher-level parietal visual areas not previously reported to contain somatosensory representations; Huang et al. 2012). There were also palatal- and retroflex-related patches in the left inferior frontal gyrus (IFG), and responses in the middle insula extending into the circular sulcus, just anterior to the expected location of S-II (Supplementary fig. 1); however, these responses did not survive at more conservative cluster-corrected thresholds (*P* < 10^−5^, corrected—see Fig. 1). Patches associated with retroflex to velar stops were observed in left lateral auditory cortex, as well as velar stop responses in the posteriormost planum temporale; there was one homologous, similarly organized patch of retroflex to velar stop representation midway along the right superior temporal gyrus. In general, these patches of activation were not as consistent across individuals as the somatomotor maps and showed a narrower phase spread. The activation patches in IFG and insular regions may reflect articulation of phones that are non-native to English. While familiar to our phonetically trained subjects (and practiced before the study by all subjects), the non-native retroflex and palatal stops recruited regions of IFG and insula that have been observed in previous block design studies contrasting non-native and native articulation (Moser et al. 2009); we note however that the responses we saw in these regions were nonrobust at more conservative cluster-corrected thresholds. Activation patches in perisylvian and superior temporal regions may reflect auditory monitoring by participants for the less audible phones (e.g., velar stop), or possible auditory template matching during articulation (but see Agnew et al. 2013).

Verifying the robustness of the group-level average map, we employed a more conservative initial threshold (*P* < 0.01), with a cluster-correction per hemisphere to *P* < 0.001 (cluster size: 86 mm^2^) (Fig. 1, dashed trace). We found that the more conservative initial threshold preserved the majority of the map across precentral and postcentral gyri, and central sulcus (dashed trace). As noted above, we found only small discontinuities in the map at left hemisphere postcentral gyrus (ventral to the bilabial mapping), and at right ventral central sulcus (between the velar and palatal mappings), at the more conservative initial threshold.

Comparison of articulotopy with R_1_-based group cortical myelination map (Fig. 1*b*). As a way of estimating which cortical areas contained somatotopic maps of the articulators, we overlaid the group articulotopy maps with a group mean quantitative R_1_ (1/T_1_) map from Sereno et al. (2013) whose presumptive myelination patterns were well aligned with cortical areal boundary estimates from retinotopic mapping of visual areas performed in the same participants. Using these myelin maps, we estimated the boundaries of motor areas 4 and 6, and somatosensory areas 3a, 3b, 1, and 2 based on homologies with human and nonhuman primate postmortem studies (Sherwood et al. 2004; Glasser and van Essen 2011; Sereno et al. 2013; Lutti et al. 2014; Sherwood and Hof, 2007; Petrides et al. 2005; van Essen et al. 2014; Kumar et al. 2016). We also estimated the posterior extent of areas 1 and 2 using maxima in R_1_ gradients as suggested by Glasser and Van Essen (2011).

These comparisons suggested the systematic mapping of articulatory responses extended through area 4 anteriorly, but with little sign of systematic mapping at a group level in secondary motor areas. By contrast, somatotopic mapping extended posteriorly though areas 3a 3b, 1, and 2 and past the gradient-maximum-defined posterior border of area 2 into higher-level somatosensory areas.

Comparison of 4-subject “articulotopy” with articulation-versus-baseline activation contrast (Fig. 3*a*): In order to estimate the extent to which cortical regions involved in articulation showed somatotopically mapped responses, we compared an average phase-encoded articulator map to a simple comparison of articulation versus resting baseline, both performed by a subset of the 4 participants contributing to the 10-subject average (see Methods). Articulating compared with rest activated a large patch of cortex encompassing approximately the lateral half of the pre and postcentral gyrus and intervening central sulcus, with a medial extension that partly overlapped the “hand knob” on the precentral gyrus (Fig. 3*a*, middle). Multiple lateral auditory regions along the superior temporal gyrus were also activated.

Comparing the 2 maps, almost all of the cortex activated posterior to the central sulcus also preferentially responded to a particular combination of articulators. This held true even when the statistical threshold of the articulation-versus-baseline map was set very liberally (vertexwise *P* < 0.01). The block-design activation extended more superiorly and inferiorly than the place-of-articulation-specific activation, particularly on the lateral-most aspect of the precentral gyrus (Fig. 3*a*, top). The retroflex response in the phase-encoded map also extended slightly beyond the bounds of the block-design activation posteriorly at postcentral sulcus.

Comparison of alveolar- and glottal-stop-evoked activation (Fig. 3*b*): We were surprised by the lack of clear preferential activation for glottal stops compared with other articulation positions (the phase-encoded method only reports the stimulus that evokes highest response relative to all other stimuli in a voxel, not the only stimulus responded to by that voxel). Therefore, to confirm that glottal stops were capable of evoking detectable activation, we ran another block design experiment with the same subset of 4 subjects where we pseudorandomly alternated blocks of glottal stops, alveolar stops, and baseline fixation. Articulating glottal stops recruited much of the large patch of somatomotor cortex shared by all articulators (Fig. 3*b*); a direct comparison with alveolar activation showed very similar results to the phase-encoded maps—there was no indication of preferential activation for glottal stops above all other positions, but alveolar stops evoked greater activation than glottal ones in the swath of cortex where there was preferential activation for alveolar, retroflex, and palatal stops (particularly in the more lateral (inferior) aspects of the map; Fig. 3*b*). Notably all of these articulations involve movement of the tongue and contact of the tongue with the upper surface of the mouth, in contrast to both bilabials and velars.

**Figure 2.**
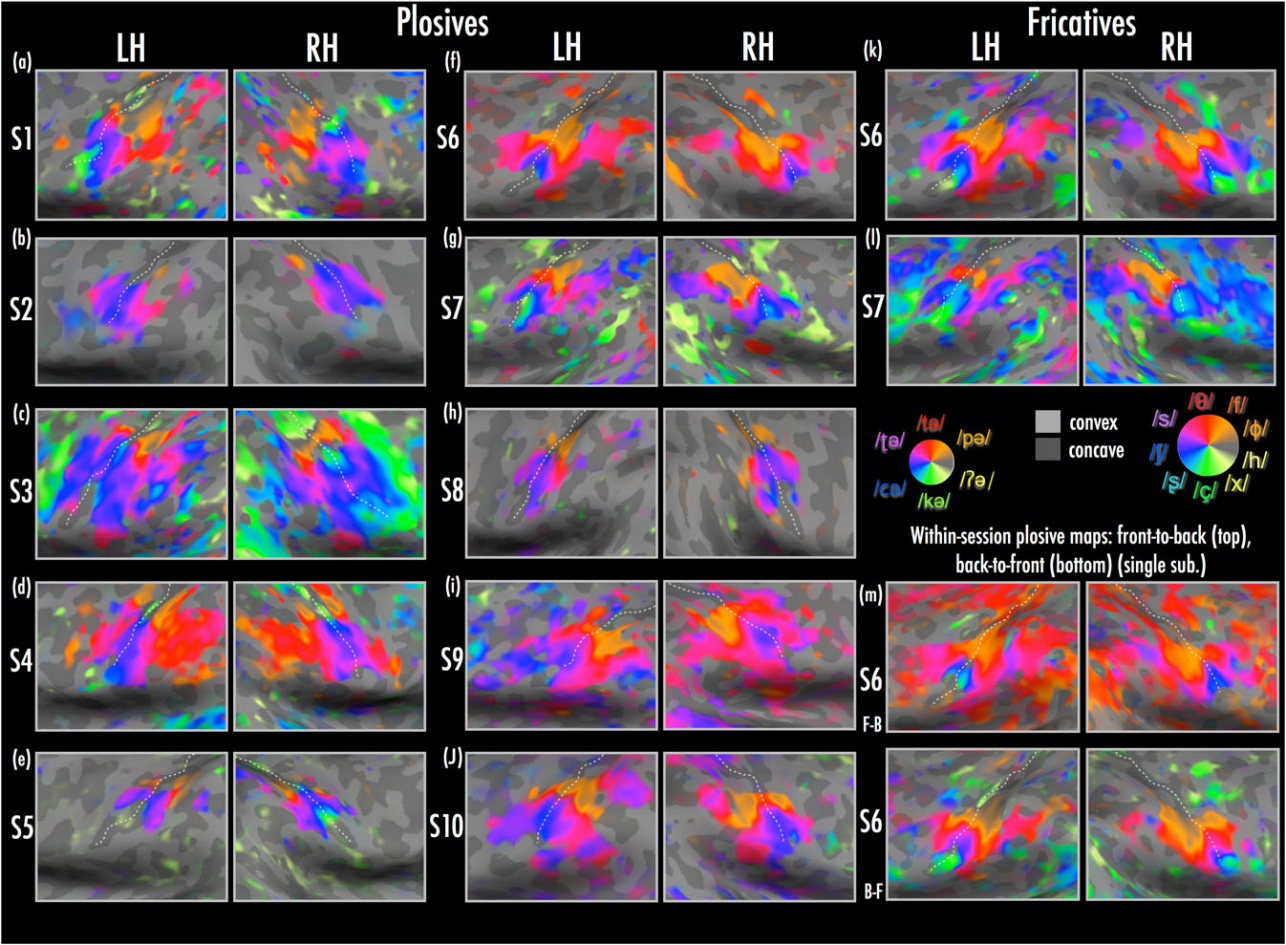
Close-ups of individual participants’ articulator maps across inflated left and right hemispheres (lateral to ventral view). Individual subjects are displayed according to scaling of complex *F* statistics using a sigmoid function with midpoint of 3.03 and slope of 1.5 (reflecting the subject-level statistics of Huang et al. 2012); *F*(2, 255) = 3.03, *P* < 0.05, uncorrected. Single-subject maps are shown for: plosive articulation condition (main experiment) (*a*–*j*); fricative articulation condition (*k,l*); within-session front-to-back and back-to-front single runs for S6, from main plosive articulation experiment (*m*) (*note*: the statistical threshold for these single runs was reduced to a midpoint of 2.2 for ease of visual comparison with the four-run average; single-run maps were robust at *F*(2, 255) = 3.03, *P* < 0.05, uncorrected).

Individual participants (Figure 2*a–j*). While the average map shows a consistent, roughly dorsal-to-ventral mapping of front-to-back sites of articulation across participants, there were individual differences in these maps in extent, but also in the internal map organization for certain articulators. Microelectrode mapping experiments in nonhuman primates at higher densities than is possible here suggest that interindividual differences should be expected.

There was activation related to bilabial production of /pə/ in somatosensory cortex in all participants, including presumptive areas 3a, 3b, 1, and 2 (see comparison with individual R_1_ maps below), and S-II (Huang et al. 2012). The activation swath occupied the central sulcus just lateral to where it joins the middle frontal gyrus, approximately at the position where Huang et al. (2012) reported their dorsal-most patch related to lip stimulation. There was also bilabial activation on the anterior bank of the central sulcus in subjects 4 and 10.

The most consistent activation for articulating alveolar /tə/ was typically just lateral to that for bilabials, and across subjects tended to extend laterally in a very thin arc down the anterior and posterior banks of the central sulcus. S1, 6, 7, and 9 also showed indications of maximal activation for alveolar articulation in secondary somatosensory regions, typically abutting bilabial-related activation. With exception of S1 and S4, in all subjects who showed alveolar-related activation in posterior somatosensory areas, the thin “alveolar arc” was surrounded inferiorly and laterally by a much larger swath of activation maximal for retroflex stops (Inspection of native-space EPI data in individual subjects showed that the thin alveolar arc was not due to phase smoothing artifacts). A total of 5 subjects (S3, 6, 7, 9, and 10) showed maximal /ʈə/ retroflex-induced activation extending into posterior somatosensory regions, sometimes interleaved with bilabial or alveolar maxima, but generally inferior to these representations. However, in some participants (S1, 4, 6, 7, 9,10) there was a separate retroflex-induced patch of activation posterior and somewhat dorsal to that for bilabials and alveolars. Retroflex-evoked maxima in the remaining subjects were mostly limited to the anterior and posterior banks of the central sulcus.

In all subjects, there was a band of /cə/ palatal-evoked activation within (and inferior to) the inverted “U” of retroflex activation descending into the central sulcus. However, some subjects (S1, 3, 4, 5) also showed more medial palatal activation within the central sulcus in one or both hemispheres, lying medial to or nested within retroflex maxima. Finally, several subjects showed velar /kə/ activation just inferior to the main palatal activation band in at least one hemisphere, typically situated in the base of the central sulcus or within the subcentral gyrus. In the subjects (S1, 3, 4, 5) with a medial palatal activation band, there was also a velar-related maximum just medial to the palatal activation, forming a rough mirror image arrangement within the central sulcus (velar-palatal-retroflex-palatal-velar). There was little evidence of a separate glottal representation in the phase-encoded data (as in the group average data). Some subjects (in particular S1 and 3) showed widespread activation in motor and somatosensory areas during velar and glottal stops, but this may have been due in part to the difficulty some subjects had in repeating these articulations while lying supine, and controlling breathing and airflow.

**Figure 3.**
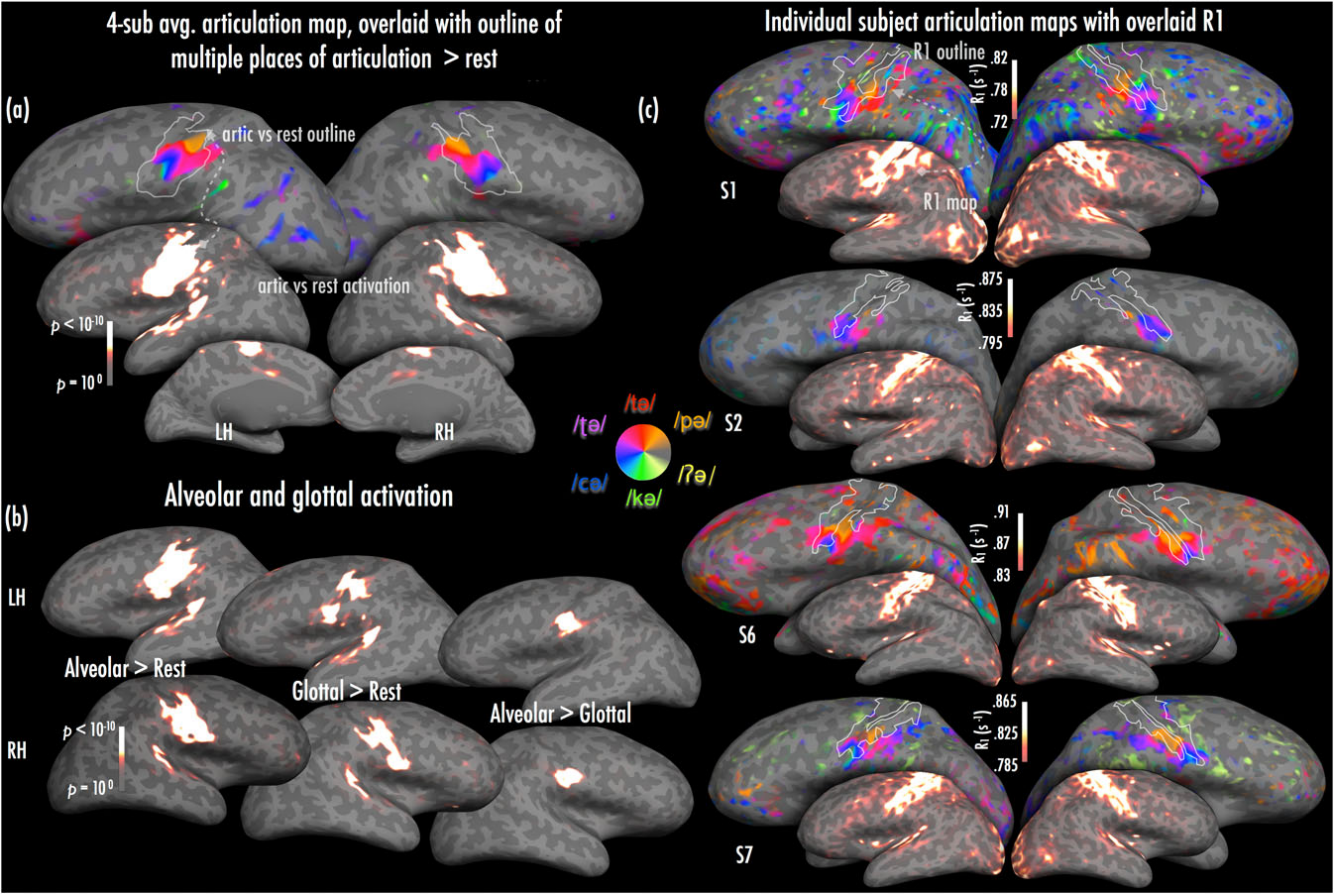
(*a*) Articulation map from subset of 4 subjects (S1, S2, S6, S7) shown in top and thresholded/colorscaled as in Figure 1, with inset heatscale statistical maps displaying block design activation for all voiceless stops > rest in the same subjects (displayed at statistical midpoint corresponding with *P* < 10^−6^, uncorrected). The contour on the articulation map was traced at this statistical midpoint, and shows the main activation cluster in somatomotor regions. (*b*) Activation (heatscale) statistical maps for the same subjects, showing contrasts of alveolar stops > rest, glottal stops > rest, and alveolar stops > glottal stops (all with statistical midpoint corresponding with *P* < 10^−6^, uncorrected). (*c*) Individual subject articulator maps (thresholded as in Fig. 2) with individual subject's R_1_ traced on each map from the inlaid heatscale R_1_ overlays.

Comparison of articulator and myelin maps within individual participants: To further site our articulator maps with respect to myeloarchitectonic patterns marking different somatosensory and motor areas, we compared phase-encoded maps to R_1_ maps at the single-subject level (Fig. 3c). As with the comparison between average phase-encoded articulation maps and average R_1_ maps, single subject comparisons showed that articulation maps covered R_1_-defined area 4, and tended not to extend anteriorly to secondary motor areas. The exception was subject S6, where the retroflex map extended to presumptive area 6/44 in the left hemisphere. Again recapitulating the group data, in postcentral cortex, subjects S1, S6, and S7 showed clear extension of the articulator maps beyond area 3a and 3b, into secondary somatosensory regions in the postcentral sulcus. The maps in one subject (S2), by contrast, were largely confined to areas 3a and 3b over both hemispheres.

Comparison of maps across manner of articulation (Fig. 2*k,l*): In a separate condition paralleling the main experiment with articulated stop consonants, S6 and S7 were scanned while repeating 9 different unvoiced fricatives that, as in the main experiment, varied in place of articulation from labial to glottal (Fig. 2, right inset). In contrast to the main experiment with articulated stops, here the oral articulators (tongue and lips) moved much less. The place of articulation was defined by changing the narrowed location in the orophayrngeal cavity, which results in localized turbulent airflow. The resulting somatosensory stimulus is somewhat similar to skin stimulation via gentle airpuffs (Sereno and Huang, 2006; Huang et al. 2012). This was a challenging condition (particularly for S7) due to the increased demands on breath control due to greater overall airflow, which in turn resulted in increased drying of the mouth, salivation, and swallowing. Nevertheless, the stop-evoked articulation location mapping is quite strikingly recapitulated in the fricative maps in both subjects, anterior and posterior to the central sulcus (Fig. 2*k,l*). Indeed, an important consideration here is that the airflow and nature of contact between the articulators—key fundaments of speech—differ somewhat between the plosive and fricative conditions. Nevertheless, the maps that we observed suggested that changes in the place of articulation did lead to similar representations of articulator positioning in both conditions.

Comparison of maps within and between sessions (Fig. 2*m*). Finally, we inspected maps for stability, both across sessions, and within a single session. Examining front-to-back and back-to-front runs at a single-subject level suggested high correspondence in map location and articulator representations across runs in the same session (compare Fig. 2*m*, upper [front-to-back] and lower [back-to-front] panels). Comparison of these front-to-back and back-to-front orders with the fricative map further suggested strong correspondence across manner of articulation, and indeed, across different scanning sessions conducted on separate days (plosive and fricative maps were collected in different sessions more than 1 year apart).

## Discussion

Using phase-encoded fMRI mapping methods, we demonstrate topological maps of articulation both in face motor and somatosensory cortex. Building on previous work (Brown et al. 2008, 2009; Meier et al. 2008), we found especially robust mapping of the anterior and middle places of articulation of the vocal tract. Similar responses occurred in most participants for production of plosives and fricatives at labial, alveolar, retroflex, and palatal places of articulation, with homologous responses in the group average. In contrast, production of speech phones at the most posterior places of articulation yielded less reliable responses across subjects; variable and more limited velum- and larynx-specific mapping was found at individual and group level. The relative under-representation of posterior articulation positions was unlikely to reflect inaccurate or irregular articulatory performance, since most of our subjects had extensive training in phonetics and all were well-practiced at the tasks. Additional conditions—both phase-encoded and block design—demonstrated similar map emphasis on more anterior articulation positions using a different manner of articulation, and similarly small unique representation of the posterior glottal position.

Present results broadly agree with previous studies of somatosensory and motor representations of the human vocal tract. Brown et al. (2008) found a superior to inferior pattern of oral representation across M-I, with lip activation located superior to tongue (see also Pulvermüller et al. 2006; Meier et al. 2008). Brown et al. (2008) also reported larynx areas in inferior-medial central sulcus and dorsolateral premotor cortex (i.e., superior to lip representation), active both during schwa phonation and adduction of the vocal folds (see also Brown et al. 2009). We found limited evidence of laryngeal mapping at the group level, but did note small responses in lateral regions of central sulcus of 2 subjects (Fig. 2*a* [LH] and *g*). Furthermore, we ran an additional block-design condition, and compared glottal stop production (i.e., laryngeal place of articulation) with alveolar stop production; however, this contrast failed to yield consistent activation in 4 subjects (cf. Brown et al. 2009). In further contrast to the results of Brown et al. (2008, 2009), a recent magnetoencephalography study (Miyaji et al. 2014) showed a more inferior central sulcus activation related to laryngeal (arytenoid) airpuff stimulation (visually similar to that evoked by tongue movement in Meier et al. 2008), with a more superior focus for tactile stimulation of the right buccal mucosa, that was in turn inferior to primary activation related to hand stimulation. Future phase-encoded investigations with alternative stimulation methods may allow us to determine if laryngeal place of articulation can be mapped across multiple subjects.

A strength of the present results is the evidence of reproducibility of the place of articulation maps, within a single session (phase-averaging within-session showed front-to-back and back-to-front runs were highly similar) and across sessions (comparing plosive articulation to fricative articulation in the same subject). In particular, phase-averaging of within-session runs would lead to cancellation of phase if the articulation response had been inconsistent across cycle directions, which would lead to little evidence of robust subject-level average articulator maps (our subject-level findings point to the contrary). With respect to between-session replication, fricative, and plosive scanning sessions for that subject were conducted more than a year apart, yet still yielded strikingly consistent average maps. The similarity of maps across the plosive and fricative conditions suggests that despite the differences in air flow and articulator dynamics between those conditions at each place, somatomotor functional representations appear to reflect the positions and arrangement of the articulators.

Our results extend the results of recent clinical ECoG investigations of speech phone articulation (Bouchard et al. 2013; see also Cheung et al. 2016). These studies have demonstrated similar arrangement of somatomotor articulator maps for production of plosives varying in place of articulation (i.e., increased response amplitudes at dorsal vs. inferior somatomotor electrode locations, for bilabial versus velar stop production, respectively; Bouchard et al. 2013). Our use of cortical surface-based functional mapping methods allowed us to explore somatomotor representations during speech throughout the fundus of the central sulcus, covering the complete extent of area 4 and areas 3a/b (Glasser and van Essen 2011). Similar map resolution is currently not achievable with ECoG methods because electrodes are typically not placed deep in sulci. Our results also extend ECoG findings to a nonclinical sample, and demonstrate that somatomotor representations of the articulators can be mapped via noninvasive, phase-encoded functional neuroimaging. A remaining question concerns the specific computational processes involved in sequencing articulator movements, which form a requisite mechanism of speech production (Guenther 2006; Bohland et al. 2010; Cogan et al. 2014; Simmonds et al. 2014). While our present findings do no speak directly to the computational components of planning and sequencing of articulator movements, more detailed topographical maps of vocal tract representations nevertheless move us a step closer towards improved understanding of those mechanisms across somatomotor cortex.

An important question raised by the present findings is the extent to which maps reflect somatosensory versus motor activity. Our paradigm did not allow us to disentangle the relative contributions made to the maps by primary motor afferents versus mechanoreceptive input to M-I (Huang et al. 1989a; Matyas et al. 2010), M-I corticocortical projections to S-I (Kinnischtzke et al. 2013; Cerkevich et al. 2014), or direct ascending somatosensory input to M-I via dorsal column inputs to VL thalamus. The role of somatosensory input in skilled motor performance and learning has been well-documented (Pavlides et al. 1993; Wu et al. 2005). It is known that tactile input via electrical stimulation of median nerve yields functional activation in both human M-I and S-I (Spiegel et al. 1999) and that peripheral nerve stimulation increases excitability of cortical motorneurons (Kaelin-Lang et al. 2002). Investigations in macaques have shown that stimulation of oral surface cutaneous receptors elicits spiking from M-I neurons representing movement of jaw, tongue, and other facial musculature (Huang et al. 1989a, 1989b; Murray and Sessle 1992a). Models of primate motor cortex suggest that motor representations across the posterior extent of M-I may be arranged in a more “primary-like” somatotopic fashion (akin to S-I) than anterior M-I regions (Graziano and Aflalo 2007). Our group average articulation map showed a broadly parallel arrangement across pre and postcentral gyri, and central sulcus. This likely reflects the close interdependence between M-I and S-I representations, although we note that maps did extend posteriorly beyond S-I (see below).

Although we aimed to map representations of the vocal tract within somatomotor regions, we anticipated that maps might emerge outside of these areas. We found relatively small responses in left perisylvian and bilateral superior temporal regions for articulation of palatal and velar stops. However, there was little evidence of graded articulator maps in regions implicated in speech production networks: IFG, supplementary motor area (SMA), or anterior insula (Ackermann and Riecker 2004; Guenther 2006; Sörös et al. 2006; Loucks et al. 2007; Riecker et al. 2008, 2006). The relatively simple tasks our participants completed (repetitively producing a single speech phone at each place of articulation), may not have been sufficiently demanding to recruit other cortical regions (Murphy et al. 1997; Lotze et al. 2000). For instance, increasing the rate of utterance production yields greater activation in SMA and insula (Riecker et al. 2006). SMA is also more active during production of alternating speech phones versus sustained phonation alone (Loucks et al. 2007). Further, production of low frequency-of-occurrence, complex onset (i.e., CCV) syllables recruits IFG to a greater extent than production of simpler (i.e., CV), high frequency syllables (Riecker et al. 2008). Thus, our results do not rule out the possibility of articulation-specific maps beyond somatomotor areas; maps could emerge in regions such as IFG or SMA when more complex or variable sequences of articulator movements are produced. A recent phase-encoded mapping study that used auditorily cued movements demonstrated 2 full body maps within SMA (Sood and Sereno 2016). However, since that study more coarsely covered regions from head to toe, it is possible that maps of place of articulation remain to be found in SMA (further to Peeva et al. 2010).

The relative similarity of cortical responses when different subclasses of speech phones were articulated holds implications for somatomotor representations of speech production. Voiceless stops and fricatives produced at comparable places of articulation revealed broadly similar maps across 2 subjects (compare Fig. 2*f* and *k*;* g* and *l*). It appears then that somatomotor maps reflect changes in place of articulation, with potentially less clear distinction between contrastive locations (see Bouchard et al. 2013; further to Correia et al. 2015). This suggests that the relative position and movement/contact of the articulators (i.e., articulatory place) could be more critical to somatomotor maps than manner of articulation (Cheung et al. 2016; Chang et al. 2009).

Previous anatomical and electrophysiological investigations using nonhuman primates have shown clear correlations between myelination and somatomotor maps (Jain et al. 2001; Cerkevich et al. 2014). Our work similarly showed considerable overlap between articulation location maps and in vivo metrics of cortical myelination (i.e., R_1_ = 1/T_1_; Sigalovsky et al. 2006; Dick et al. 2012; Sereno et al. 2013). Previous investigations have used maps of cortical myelination to delineate the boundaries of cortical regions not definable by functional activation alone (Glasser and van Essen 2011; Dick et al. 2012; Sereno et al. 2013). Present results suggest that functional maps of place of articulation overlap with cortical myelin markers in lateral to ventral precentral areas, and lateral postcentral gyrus (Figs 1 and 3*c*); however, our phase-encoded maps extended beyond the bounds of highest-intensity R_1_ into the postcentral sulcus (i.e., secondary somatosensory regions). This may suggest that secondary somatosensory regions known to comprise multiple re-mappings of the body surface (Huang et al. 2012) may additionally be involved in representing articulator position and contact during speech.

Our maps of the production of complex oral movements involving a variety of muscles parallel data from previous studies of motor cortex and parietal cortex, which have suggested partial organization of these regions around ethological movement categories (Graziano et al. 2002a; Graziano and Cooke 2005; Stepniewska et al. 2009). Within a particular movement category, there is often topological mapping of motor targets, for example, locations in hand-accessible extrapersonal space. The suggestion is that rather than individual points in motor cortex representing isolated muscles, individual points may instead represent the particular combination of muscle contractions required to move the hand to a particular position, and that nearby points in the cortex code for movements to nearby positions in target space (Graziano et al. 2002a, 2002b, 2005; Overduin et al. 2012). The map of place of articulation we found in motor cortex might be another example of this type of organization.

In sum, the present study provides evidence for topological somatomotor representations of the human vocal tract, with a greater map emphasis on anterior versus posterior places of articulation. We demonstrate similarity of mappings across different manners of articulation, as well as overlap between articulator and R_1_ myelin proxy maps in pre and postcentral regions. Our findings show that speech phone articulation combined with phase-encoded mapping methods can be used to naturalistically engage many of the sensory surfaces and muscles of the vocal tract in a repeatable and noninvasive manner. These results may inform future clinical investigations seeking to understand possible variations in the organization of articulator maps in speech pathologies.

## Supplementary Material

Supplementary material can be found at *Cerebral Cortex* online.

## Funding

Medical Research Council (NIA G0400341 and G0700399), The Royal Society (RG081218), Royal Society Wolfson Research Merit Award (NIH RO1 MH 081990), and European Commission Marie Curie Early Career Training Network (MC-ITN-264301 TRACKDEV). The Wellcome Trust Centre for Neuroimaging is supported by core funding from the Wellcome Trust (091593/Z/10/Z).

## Supplementary Material

Supplementary Data

Supplementary Data
