# Supplementary material for: Functional and Quantitative MRI Mapping of Somatomotor Representations of Human Supralaryngeal Vocal Tract
Source: Cereb Cortex. 2017 Jan 9;27(1):265–78. doi: 10.1093/cercor/bhw393 (PMC5808730; doi:10.1093/cercor/bhw393)
Supplement: Supplementary Data [file carey_et_al_supp_legend.docx]

**Supplementary Figure 1:** Group average articulator map with more liberal threshold than Figure 1 (initial threshold *p* < 0.05, cluster size 134 mm^2^, hemisphere-wise corrected threshold *p* < 0.05). All other figure specifications as per Figure 1.
