# Supplementary figures and images for: Functional and Quantitative MRI Mapping of Somatomotor Representations of Human Supralaryngeal Vocal Tract
Source: Cereb Cortex. 2017 Jan 9;27(1):265–78. doi: 10.1093/cercor/bhw393 (PMC5808730; doi:10.1093/cercor/bhw393)

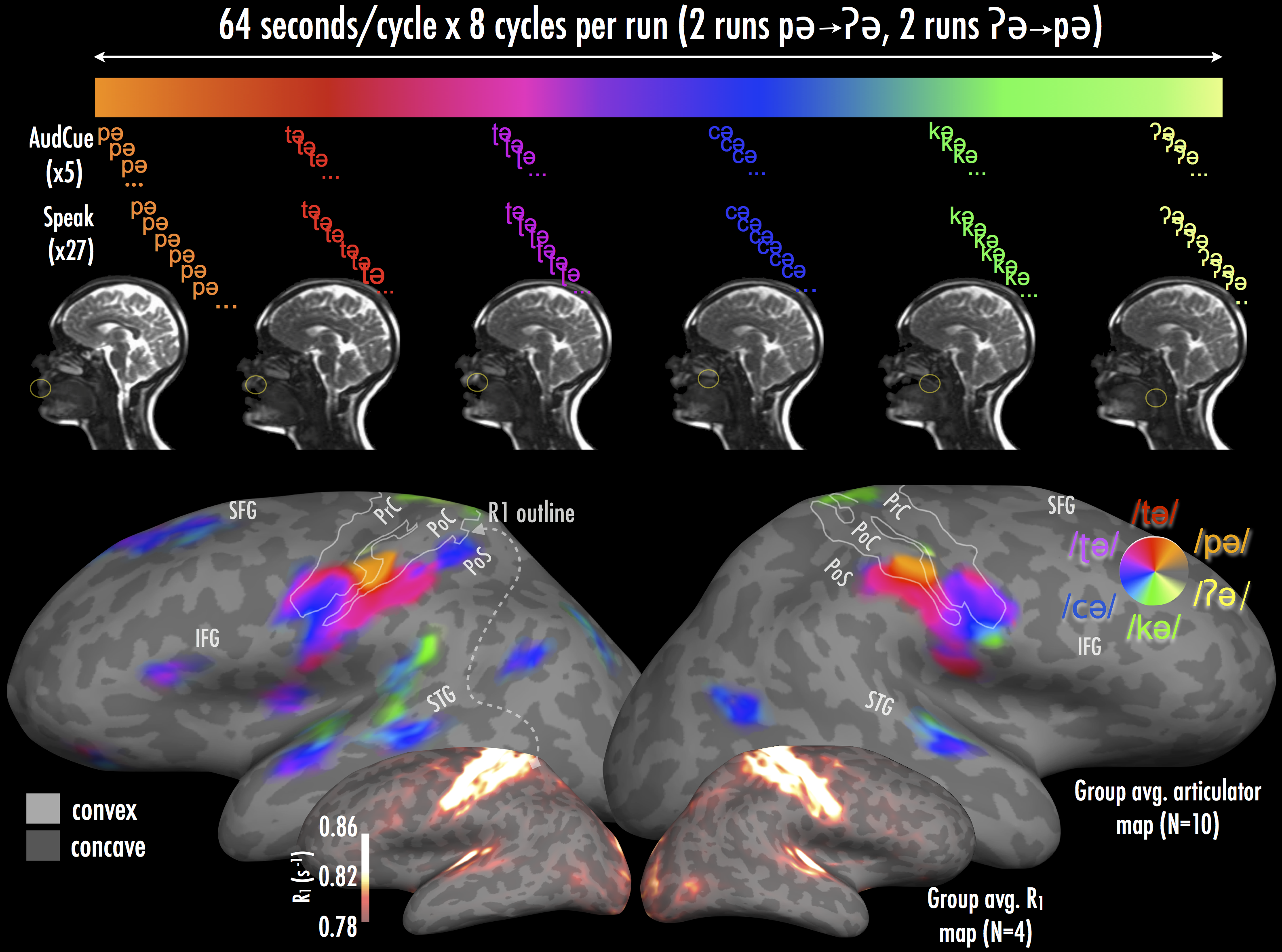

Supplement: Supplementary Data [file figures_carey_et_al_mri_of_vocal_tract_somatomotor_representations_revised_suppl_fig1.png]
